# Rheumatoid Arthritis and Familial Mediterranean Fever or Sacroiliitis Accompanied by FMF

**DOI:** 10.1155/2013/636713

**Published:** 2013-12-22

**Authors:** Ali Şahin, Alparslan Yetişgin, Mehtap Şahin

**Affiliations:** ^1^Division of Rheumatology, Sanliurfa Education and Research Hospital, 63100 Sanliurfa, Turkey; ^2^Division of Physical Therapy and Medicine, Sanliurfa Education and Research Hospital, 63100 Sanliurfa, Turkey; ^3^Physical Therapy and Medicine Unit, Sanliurfa Education and Research Hospital, 63100 Sanliurfa, Turkey

## Abstract

The coexistence of rheumatoid arthritis (RA) and familial Mediterranean fever (FMF) has been rarely seen in case reports in the literature. Herein, we wanted to present a patient who had been followed up and treated as RA, but on investigation we concluded that he really had FMF and its joint complaints associated with sacroiliitis. Recovery was achieved by etanercept administered as if he was an RA patient.

## 1. Introduction

Familial Mediterranean fever (FMF) is an autosomal recessive, autoinflammatory disease characterized by recurrent self-limiting attacks of fever, arthritis, and serositis [1]. Mutations in the pyrin-encoding gene Mediterranean fever (MEFV) on chromosome 16p13.3 are responsible for clinical findings and different phenotypical features [2, 3]. Pyrin proteins affected by these mutations alter inflammatory processes via some cytokines, especially interleukin-1*β*. Inflammatory processes can cause arthritis (in any joints, including the sacroiliac joint), fever, serositis, and erysipelas-like skin lesions. Nevertheless, phenotypical variations may be seen among patients having the same genotypes [2, 3]. Spinal (i.e., sacroiliitis) and peripheral joint (i.e., knee arthritis) involvements of FMF can be resembling spondyloarthropathies (SpA) features [4]. In addition, it has been notified that sacroiliitis could be seen in 7% of Turkish FMF patients [4]. M694V mutation can be encountered significantly higher frequencies in FMF patients with sacroiliitis [4]. Recently, antitumor necrosis factor (TNF) agents have been frequently used to produce a clinical response in patients with resistance to colchicine therapy [4–6].

The coexistence of rheumatoid arthritis (RA) and familial Mediterranean fever (FMF) has been rarely seen in case reports in the literature [6, 7]. Herein, we wanted to present a patient who had been followed-up and treated as RA, but on investigation we concluded that he really had FMF and its joint complaints associated with sacroiliitis. Recovery was achieved by etanercept administered as if he was an RA patient.

## 2. A Case Report

A 45-year-old man had initially been admitted for arthritis in each knee in 2005. He was diagnosed as RA in another health center and treated with sulfasalazine (SSZ), methotrexate (MTX), and prednisolone. However, he did not use this therapy regularly. He suffered from abdomen, back, and low-back pain. His laboratory test results were as follows: erythrocyte sedimentation rate (ESR) 57 mm/h, C-reactive protein (CRP) 3.91 (0–0.8) mg/dL, and RF 21.1 IU/mL in 2010. He had elevated AST, ALT, and ALP levels and HBV (−), HCV (−), and HIV (−). His urine analysis, endoscopy, and colonoscopy investigations were normal. He was administered intra-articular steroid injections (to each knee) and steroids orally to treat the arthritis, elevated ESR, and CRP. He started treatment with MTX, SSZ, and hydroxychloroquine (HCQ) for maintaining relief from knee arthritis attacks and to maintain normal hepatic functions in 2011. The low-back pain and stiffness complaint started in February 2012. His lumber magnetic resonance imaging (MR) showed scoliosis. He again had elevated ESR, CRP, and knee arthritis but anti-CCP: 7 (0–17 U/ML) (normal), RF: 8.5 IU/mL, and brucella negative. For this reason, leflunomide was added to his therapy.

**Figure 1:**
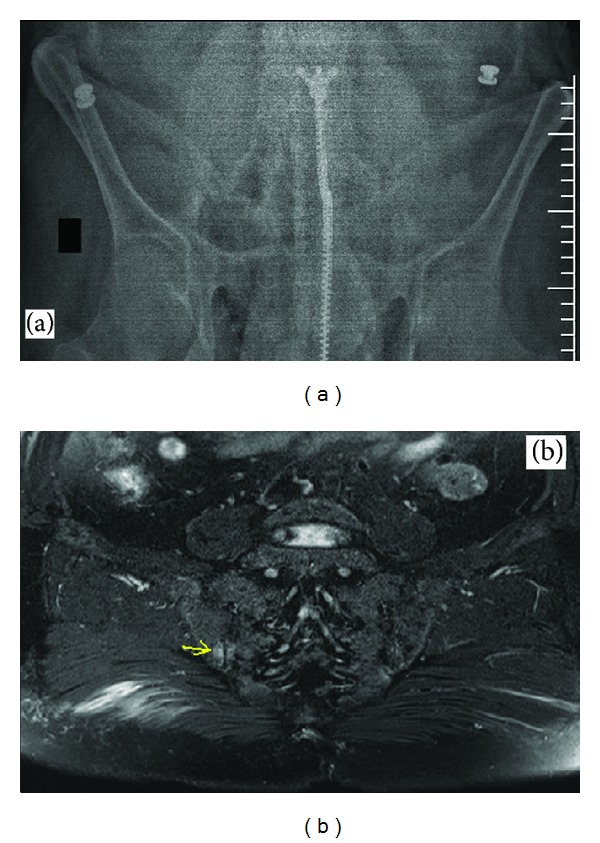
(a) Plain sacroiliac joint X-ray showing irregularities in each joint space (apparent in the right). (b) Evident bone marrow edema area in right side on sacroiliac magnetic resonance (MR) (yellow arrow).

On his followup, we observed no response to the treatment and made a tuberculin skin test (TST) with purified-protein derivative (PPD). His PPD was measured as 5 mm. We started to administer etanercept as an anti-TNF therapy in January 2013. We achieved a good response at the 3rd month of treatment, but he was again admitted to our hospital for low-back pain in May 2013. We carried out a sacroiliac X-ray to look for spondyloarthropathy. Despite the fact that the X-ray was inconclusive (Figure 1(a)), we planned sacroiliac MR and HLA-B27. On his sacroiliac MR, bone marrow edema in the posterior region of each sacroiliac joint with T2A signals and contrast enhancement (Figure 1(b)) was detected. This was interpreted as indicating acute sacroiliitis. In addition, a MEFV gene mutation test was planned for recurrent arthritis attacks and the sacroiliitis because of HLA-B27 negativity. E148Q (at 2nd exone) and A744S (at 10th exone) heterozygosity was detected; and colchicine was added to his treatment protocol. Lumber total *T* score L1–L4 = −4.5/L4 = −4.9 and femur total *T* score −2.4 on dual X-ray absorptiometry (DEXA) were measured. Bisphosphonate therapy was also commenced. His urinary protein excretion was 172 mg/24 hrs. As a result of the tests, it was thought that he had FMF; and its joint manifestations (knee arthritis attacks) and sacroiliitis were attributed to FMF rather than RA. Informed consent was obtained from the patient.

## 3. Discussion

The relationship between MEFV mutations and some rheumatic diseases such as juvenile idiopathic arthritis (JIA), RA, ankylosing spondylitis, systemic lupus erythematosus (SLE), Sjögren's syndrome, and polymyositis has been detected [6, 8–12]. It has been suggested that MEFV mutations can be an aggravating factor for the disease severity of RA patients [13]. In addition, RA patients can also have FMF simultaneously. Sometimes, anti-TNF therapy or other nonbiological disease-modifying antirheumatic drugs (DMARDs) using for RA symptoms therapy can mask the real clinical expressions of FMF.

The heterozygous E148Q allele in the MEFV gene may be a causative reason for a patient to be a carrier only or to have nondominant phenotypical features; thus, it can be a benign genotype. In a study from Turkey it has been shown that the E148Q allele leads to a true inflammatory phenotype [14]. In another study it was explained that M694V, E148Q, and V726A mutations occasionally could be measured in 202 patients and these had better correlations between genotype and phenotypical characteristics [15].

As in this case, if a patient has recurrent large joint arthritis attacks and shows no response to disease modifying agents, we should check our diagnosis and treatment modalities. In our case, he does not have a family history of FMF, but he was from the Mediterranean region of Turkey (Iskenderun). Initially, he had a low titer of RF positivity, but a normal range of anti-CCP. He had no small joint (i.e., MCP and PIP) involvements and deformities for this reason. However, he had been diagnosed and treated as RA from 2005 to 2013. Achieving good clinical response by etanercept is supported by the literature for FMF. However, the exciting point is that patients who have E148Q positivity can have better clinical findings than the others as we know. We wonder whether it could be due to different clinical and phenotypical reflections of the FMF genotype as a course of its nature.
